# Role of Intermediate Filaments in Vesicular Traffic

**DOI:** 10.3390/cells5020020

**Published:** 2016-04-25

**Authors:** Azzurra Margiotta, Cecilia Bucci

**Affiliations:** Department of Biological and Environmental Sciences and Technologies (DiSTeBA), University of Salento, Via Provinciale Lecce-Monteroni 165, 73100 Lecce, Italy; azzurramarg@libero.it

**Keywords:** intermediate filaments, intracellular trafficking, endocytosis, exocytosis, vimentin, membrane traffic, mitosis

## Abstract

Intermediate filaments are an important component of the cellular cytoskeleton. The first established role attributed to intermediate filaments was the mechanical support to cells. However, it is now clear that intermediate filaments have many different roles affecting a variety of other biological functions, such as the organization of microtubules and microfilaments, the regulation of nuclear structure and activity, the control of cell cycle and the regulation of signal transduction pathways. Furthermore, a number of intermediate filament proteins have been involved in the acquisition of tumorigenic properties. Over the last years, a strong involvement of intermediate filament proteins in the regulation of several aspects of intracellular trafficking has strongly emerged. Here, we review the functions of intermediate filaments proteins focusing mainly on the recent knowledge gained from the discovery that intermediate filaments associate with key proteins of the vesicular membrane transport machinery. In particular, we analyze the current understanding of the contribution of intermediate filaments to the endocytic pathway.

## 1. Introduction

Intermediate filament proteins (IFs) are a large family that constitutes one of the three components of the cytoskeleton. This family includes a wide number of proteins that, in the human genome, derive from 70 different genes. Furthermore, multiple splice variants can originate from the same gene thus increasing the variety of proteins constituting IFs [1,2,3,4]. IFs, according to their denomination, have an intermediate size (8–15 nm diameter) compared with the other cytoskeleton components, microtubules and actin microfilaments, whose diameter is 25 nm and 5–8 nm, respectively [5,6,7,8,9,10]. Moreover, IFs differ from the other cytoskeletal members for their apolarity which prevent them to be used as tracks for motor proteins [11].

Despite the wide number of IFs, all the members of this family share the same tripartite structural organization. In fact, IFs are composed by a central α-helical rod domain flanked by two non-α-helical domains. Amino acids are organized in heptad repeat pattern in the central rod domain, whereas variability in the primary structure of IFs characterizes the amino-terminal head and the carboxy-terminal tail domains. The *in silico* structural prediction of IFs suggests that the central domain is divided into four subdomains, interrupted by three linker domains (L1, L12, L2), where the heptad repeats form four sub-helices (coil 1A, coil 1B, coil 2A, coil 2B), but crystallographic studies indicate that the segments coil 2A, L2 and coil 2B constitute a continuous coiled coil domain [10,11,12,13,14]. The structure of IFs is important for self-assembly. In fact, unlike the other cytoskeleton components, intermediate filament (IF) monomers do not have any enzymatic activity, thus their assembly is based on the association of several monomers. In particular, two α-helical rods associate in parallel creating a dimer, which, in turn, associates with another dimer in an antiparallel manner, giving rise to a tetramer. Tetramers rapidly associate laterally in order to form unit length filaments (ULF), whose length is around 60 nm, and then they anneal longitudinally creating longer filaments [15]. Their assembly in mature insoluble filaments and their disassembly in soluble components (from tetramers to monomers) are regulated by phosphorylation/dephosphorylation cycles [16].

According to comparisons on the IFs primary structure in higher vertebrates, IFs have been grouped into six types or classes. Acidic and basic keratins have been catalogued into the I and II class, respectively. Class III is the most heterogeneous as it includes desmin, glial fibrillary acidic protein (GFAP), peripherin and vimentin while class IV comprises α-internexin, nestin, synemin, syncoilin and the neurofilament (NF) triplet proteins, which are classified according to their molecular weight in NF-L (light), NF-M (medium) and NF-H (heavy). Lamins belong to the fifth class and, unlike the IFs of classes I-IV that constitute the cytoplasmic IFs, compose the nuclear IFs. Class VI has been added recently and contains two lens-specific proteins, the beaded filament structural proteins, Bfsp1 (known also as filensin) and Bfsp2 (also known as phakinin or CP49) [11,17]. Keratins can form only heteropolymers in epithelial cells combining proteins of the I and II class in a 1:1 ratio [18], while type III IFs form mainly homopolymers but are able also to associate with other type III or type IV IFs. For instance, vimentin forms heteropolymeric filaments with desmin in vascular smooth muscle tissue [19], with glial fibrillary acidic protein (GFAP) in glioma cells [20] and with neurofilaments [21].

IFs expression is highly regulated during embryonic development and cell differentiation. For instance, vimentin has a wide distribution being expressed in mesenchimal cells, in leukocytes, in blood vessel endothelial cells and in some epithelial cells [22,23,24], but it is also expressed in neurons during axonal regeneration together with peripherin [25,26]. Moreover, IFs expression presents cell and tissue specificity in adult cells [27]. For instance, desmin is expressed both in cardiac and skeletal muscle cells [24], GFAP is found in astrocytes and in other glial cells [28,29] and peripherin is present in peripheral neurons and in central neurons projecting toward peripheral structures [30,31,32]. In addition, neurofilaments are expressed in mature neurons [33]. In addition, IFs expression levels are altered in pathological conditions. In fact, overexpression of keratins has been linked to several diseases [34] and alteration of neurofilament levels in specific body compartments are hallmarks of neurological disorders [35,36]. Moreover, GFAP and vimentin are upregulated in reactive astrocytes [37], whereas peripherin is increased after injury [38].

IFs are essential components of cell architecture and functions. The differences in the head and tail domains of the various IF proteins, their different expression and their post-translation modifications are essential characteristics in order to modulate a wide range of cellular functions. The first role attributed to IFs was to support cell strength and maintain tissue structure in a static manner. In fact, IFs are important in conferring resistance to mechanical and non-mechanical stress and deformation. For instance vimentin contributes to cell stiffness both in fibroblasts and chondrocytes [39,40], but it has also elastic properties and protects against compressive stress. In embryonic fibroblasts from wild type and vimentin knockout mice it has been shown that vimentin filaments regulate intracellular mechanics by localizing and stabilizing organelles in the cell and enhancing cell elastic behavior [41,42]. Another example is represented by keratins that are important for structural integrity of epithelial cells. Indeed, keratins found mainly in epithelial cells from simple epithelia modulate cell stiffness in hepatic epithelial cells [43].

Traditional and well-established roles of IFs include also regulation of the nuclear shape, structure and activity. Lamins are localized in the nuclear lamina, which is one of the three components of the nuclear envelope, together with the nuclear membrane and the nuclear pore complexes. Lamins are important for nuclear architecture and exert important anchorage functions for structural proteins of the nuclear envelope [44]. Moreover, they are responsible for the distribution of the nuclear pore complexes in the nuclear membrane [44]. In addition, a physical connection between cytoplasmic IFs and lamins through nuclear protein complexes at the nuclear envelope has been demonstrated [45,46,47,48]. Furthermore, several studies proved that a correct lamina assembly is necessary to establish DNA replication centers and that lamins are important also for the elongation phase of the synthesis of DNA [49,50]. Lamins are also able to modulate and maintain heterochromatin domains [51,52,53,54,55] and to influence DNA transcription regulating RNA polymerase II activity [56]. Moreover, they can regulate DNA repair recruiting the DNA damage response machinery [57,58,59,60] and phosphorylation of lamins is requested for nuclear envelope breakdown during mitosis [61,62,63,64]. Lamins are not the only IFs affecting nuclear activities as also vimentin filaments, together with lamins, control nuclear shape [65,66].

Interestingly, the involvement of IFs in several other biological aspects led to the discovery of a dynamic IF network with multiple functions. IFs, microtubules and microfilaments are in constant communication and are linked together thanks to proteins called cytolinkers [67,68]. Modulation of IFs leads to modification in the microtubule and microfilament networks [67,68]. Interestingly, a direct interaction between vimentin and actin has been proved and rheological studies have demonstrated that these two cytoskeletal components contribute together to cellular stiffness [69,70]. Moreover, vimentin modulates ERKs (extracellular-signal-regulated kinases) signaling [71,72,73,74], whereas keratins are involved in the regulation of cell cycle [75]. In addition, keratins and lamins have a role in apoptosis [76,77], while vimentin, keratins and nestin have an established role in cell migration and cancer [78,79,80,81,82].

## 2. Novel Roles of IFs: Involvement in Vesicular Trafficking

Endocytosis is an active process used by the cells in order to engulf and internalize in vesicular structures molecules, macromolecules, particles and fluids from the extracellular milieu at variance with exocytosis that is used in order to export out of the cells these materials using secretory vesicles. Transport in the endocytic and the secretory pathway occurs through the formation of vesicles. In endocytosis, material from the extracellular milieu is internalized by a portion of plasma membrane that will form a vesicle. This vesicle will fuse with early endosomes, compartments with tubular extensions that are localized at the periphery of the cell. Then cargoes are sorted to late endosome in order to be then delivered to lysosomes for degradation or are recycled back to the plasma membrane directly or via perinuclear recycling endosomes. The endocytic pathway is characterized by progressive acidification of endosomal organelles, formation of multivesicular bodies and late endosomes, recruitment of lysosomal hydrolases from the trans-Golgi network and, finally, degradation of cargoes. Instead, in the secretory pathway the Golgi apparatus packages macromolecules into vesicles moving towards the plasma membrane and fusing with it exporting their content outside the cell [83,84].

A complex molecular machinery controls the different steps of intracellular vesicular trafficking such as the formation of the vesicle from the donor compartment, the selection of the cargo, the movement of the vesicle, and the tethering, docking and fusion to the acceptor compartment. Rab proteins, small GTPases belonging to the ras superfamily, are key players of this machinery, being localized to specific compartments in the endocytic and exocytic pathway and regulating the different steps of intracellular vesicular transport, from the formation of the vesicle to its fusion with the target compartment [85]. Each Rab protein regulates transport between defined compartments. For instance, Rab5 regulates the first steps of endocytosis from plasma membrane to early endosomes and it is responsible for early endosomes biogenesis and maturation, controlling cargo selection, regulating early endosome motility and fusion and recruiting a number of relevant molecules necessary for endocytosis [86,87,88,89]. At variance, Rab4, Rab11 and Rab25 regulate recycling from endosomes to the plasma membrane [90,91,92]. Surprisingly, a role of IFs in vesicular trafficking has been recently discovered and several links between IFs and vesicular membrane transport machinery have been demonstrated (Table 1).

### 2.1. Endocytosis and IFs

An increasing amount of evidence demonstrates that intermediate filaments and vesicular trafficking are strictly connected and that IFs have a role in different steps of endocytosis.

First of all some reports documented interactions between IFs and Rab proteins of sorting and recycling early endosomes, suggesting that IFs might have a role in the early steps of endocytosis [100,104,106]. For instance, Rab4, localized to sorting early endosomes and endocytic recycling vesicles, has been demonstrated to interact with vimentin in Sertoli cell, seminiferous tubule and testis [90,106]. In addition, using a photocross-linking approach, Kurzchalia and colleagues found a number of cytosolic proteins interacting with Rab5 and among these proteins there were vimentin and desmin intermediate filament proteins [100]. In fact, under conditions of ATP-depletion, both actin and polymerized vimentin and desmin filaments, were recovered in the pellet with Rab5 after sucrose gradient centrifugation [100]. These interactions have not been further investigated and thus their functional meaning and their impact on the early stages of endocytic trafficking regulated by Rab5 and Rab4 are not yet known.

Notably, the role of vimentin in the regulation of intracellular trafficking has been demonstrated at different levels. First of all vimentin associates with PKCε-positive vesicles that mediate β1-integrin trafficking, and phosphorylation of vimentin, mediated by PKCε, determines dissociation from vesicles of PKCε and vimentin as a complex, thus suggesting that vimentin has a role in β1-integrin trafficking [104,113]. In fact, vimentin localization is regulated by PKCε and expression of vimentin mutants lacking the N-terminal phosphorylation sites recognized by PKCε results in the accumulation in the cytoplasm of vesicles containing PKCε and β1-integrins, and integrins are not anymore delivered to the plasma membrane [104]. Therefore, PKCε controls the association of vimentin filaments with vesicles and the delivery of endocytosed β1-integrin to the plasma membrane from the intracellular vesicles requires PKCε-dependent phosphorylation of vimentin [104]. Interestingly, Rab4 has been shown to interact not only with vimentin but also with β1-integrins and weakly with PKCε [106], thus recycling to the plasma membrane of β1-integrin containing vesicles could be regulated by Rab4 through vimentin and PKCε. Further work is needed to test this hypothesis.

Notably, several reports document also a strong connection between IFs and late endocytic trafficking and most of them are focused on vimentin filaments. First, it has been demonstrated that vimentin interacts both with Rab7a and Rab9 [95,99]. Rab7a regulates transport from late endosomes to lysosomes and it is important for lysosome, phagolysosome and autolysosome biogenesis [114,115,116,117]. Biogenesis of lysosomes is regulated also by Rab9, which has a role not only in transport from trans-Golgi network to late endosomes but also in defining late endosomes morphology and localization [118,119]. Rab7a regulates assembly and organization of vimentin filaments modulating its phosphorylation state [95]. Indeed, Rab7a overexpression caused increased phosphorylation at Ser38 and Ser55 leading to increased amount of vimentin in the soluble fraction [95]. Moreover, in Niemann-Pick type C1 (NPC1) disease, a lysosomal storage disease, the lipid accumulation in late endosomes alters the interaction between Rab9 and vimentin leading to protein kinase C inhibition, hypophosphorylation of vimentin, aggregation and localization of Rab9 to vimentin filaments causing late endosomes dysfunction [99]. Therefore, the interaction between these Rab proteins and vimentin is important in determining the phosphorylation state of vimentin, thus its soluble/insoluble (filamentous) ratio affecting the correct transport of materials in late endosomes. Interestingly, it has to be noted that Rab7a interacts also with peripherin, an IF expressed only in the peripheral nervous system, and modulates its assembly [108]. Consequently, alteration of the functional interaction between Rab7a and peripherin might have a role in the onset of Charcot-Marie-Tooth 2B peripheral neuropathy, which is due to the expression of Rab7a mutants that bind more strongly to peripherin than Rab7 wt.

Another relevant interaction which proves the importance of vimentin in late intracellular trafficking is that between vimentin and AP-3 adaptor complex, which is important in the sorting of proteins to the endosomal/lysosomal system and is recruited from cytosol to membranes in an ARF1-GTP-dependent manner [98,120,121]. Lack of vimentin alters the subcellular distribution of AP-3 and lysosomes but also the levels of the lysosomal proteins LAMP-1 and LAMP-2, suggesting that IFs might regulate the trafficking of lysosomal cargoes, probably along with AP-3 [98]. Furthermore, it has been demonstrated that disruption of the vesicular membrane transport machinery, obtained by inhibiting ARF1, alters vimentin cytoskeleton [122]. The small GTPase ARF1 regulates the recruitment of AP-3 to endosomes and expression of a dominant-negative mutant of ARF1 causes the release of AP-3 adaptor protein from membranes [122]. Interestingly, in these conditions, it is possible to observe vimentin filaments retracted to the perinuclear region or forming dense clusters [122]. Notably, in addition to vimentin, also other two IF proteins, peripherin and α-internexin, interact with AP-3 adaptor complex thus possibly regulating late endocytic trafficking [98]. Importantly, IFs are able to control and determine the luminal content of vesicles in the late endocytic pathway. For instance, in fibroblast, vesicular ionic zinc is accumulated mainly in endosomal and lysosomal compartments [123,124]. Styers and colleagues demonstrated that vimentin-deficient skin fibroblasts show a reduction in the amount of acidic compartments, similar to AP-3-deficient cells, and, importantly, had a lower amount of ionic zinc in vesicular compartments compared to control fibroblasts [98]. As AP-3 regulates the trafficking of ClC3, an endosomal-lysosomal chloride channel important in the acidification of endocytic compartments [125], vimentin influence on the ionic composition of endocytic organelles might be due to its interaction with AP-3. Moreover, in vimentin-deficient fibroblasts defects in glycolipid synthesis have been revealed and these defects seem to be due to impaired intracellular vesicular trafficking between endosomes and the Golgi complex [107].

In addition to the regulation of lysosomal cargoes and the luminal content of late endosomes and lysosomes, IFs have a role in distribution and motility of late endocytic vesicles and compartments. For instance, vimentin, together with GFAP, regulates endosome and lysosome motility and endocytosis in astrocytes [96,102,103] while studies in desmin-deficient hearts suggest that desmin has a role in lysosome and lysosome-related organelle biogenesis and positioning [111]. In fact, desmin binds to myospryn, which, in turn, can interact with dysbindin, a component of the biogenesis of lysosome-related organelles complex 1 (BLOC-1), which regulates protein trafficking and organelle biogenesis [111]. Some IF proteins seem to be involved also in the positioning of other organelles such as, for instance, mitochondria [93,126,127] indicating organelle positioning as an important function of IF.

IFs are also important in the regulation of fast axonal transport. Indeed, overexpression of peripherin and silencing of neurofilament light, two components of IFs in neurons, cause alterations of axonal transport of endocytic organelles such as lysosomes [109]. In fact, in neurofilament light-deficient neurons lysosomal movements are faster in both directions while in the presence of peripherin overexpression lysosomes move faster only in the anterograde direction [109]. Furthermore, movements of lysosomes in axons, usually saltatory with frequent stops and changes of direction, are longer and more persistent both in anterograde and retrograde directions in neurofilament light-deficient neurons [109].

IFs seem to have a key role also in autophagy, a process that regulates degradation of unnecessary or dysfunctional cellular components, by affecting the formation and the content of autophagosomes. In fact, it has been demonstrated that treatment of cells with okadaic acid, a protein phosphatase inhibitor, inhibits autophagy and alters the organization of cytokeratin IF but not of microtubules [93]. Other data indicate that cytokeratin filaments in hepatocytes are dynamic and subjected to rapid phosphorylation/dephosphorylation cycles that affect autophagy [93]. Furthermore, vimentin-deficient cells had a reduced number of organelles labeled by monodansylcadaverine (MDC), a dye that can localize in multilamellar endosomal intermediates of autophagosomes, supporting the link between IFs and the content of autophagosomes [98]. Notably, the fact that vimentin is able to control the amount of LAMP-2 links vimentin to autophagy as LAMP-2 is a key factor not only for lysosomal biogenesis but also for autophagy. Indeed, it has been demonstrated that in LAMP-2-deficient hepatocytes autophagosomes accumulate and both macroautophagy and chaperon-mediated autophagy are impaired [98,128].

Altogether, these data indicate that IFs interact with key proteins involved in the regulation of early and late endocytic pathway such as Rab4, Rab5, Rab7 and Rab9 [95,99,100,106,108], although, while a strong involvement of IFs in late endocytic trafficking and autophagy has been established, the role of IFs in the early step of endocytosis is still unknown. At the level of late endosomes and lysosomes, IFs regulate organelle positioning, distribution, motility but also cargo selection and transport [98,102,109,111].

### 2.2. Exocytosis and IFs

Few evidences are available for the involvement of IFs in exocytosis. Communication between astrocytes and neurons is mediated by exocytic vesicles whose motility and fusion with the plasma membranes require different elements of the cytoskeleton [101]. Indeed, microtubules but also microfilaments are essential for directional motility of these vesicles towards the plasma membrane [101]. Notably, also IFs seem to have a key role in this process as depolymerisation of IFs causes a strong inhibition in trafficking of these vesicles and reduces the fraction of vesicles moving toward the plasma membrane [101].

IFs and in particular cytokeratins have been involved in the organization of the apical domain of the plasma membrane in single layered polarized epithelia [129]. Further work has established that lack of cytokeratin filaments impairs delivery to the apical domain of a number of proteins among which there is syntaxin 3, an important component of the SNARE vesicular transport machinery [94]. Syntaxin 3 is involved in the fusion of exocytic vesicles with the apical domain of plasma membrane in enterocytes and it is mistargeted when cytokeratin 8 is missing thus confirming a role of IFs in the regulation of exocytosis [94].

Recently, it has been discovered that peripherin interacts with SIP30, a neuronal protein involved in SNARE-dependent exocytosis [110]. This interaction alters SIP30 and SNAP25 subcellular distribution while SIP30 is able to affect peripherin assembly [110]. Thus, peripherin, through the close functional association with SIP30, controls the exocytic pathway in neuronal cells [110]. Moreover, two-hybrid data have indicated that a number of putative peripherin interactors are vesicular trafficking proteins. Indeed, among putative peripherin interactors there are the SNAP associated protein Snapin, the 6A subunit of the trafficking protein particle complex involved in vesicle transport during the biogenesis of melanosomes, the Cplx2 complex containing SNAP25, VAMP2 and Syntaxin1A and involved in synaptic vesicles exocytosis, and several others [110]. However, further work is needed to validate all these interactions and to investigate their functional meaning. In addition, it has been demonstrated that vimentin binds to SNAP23, a ubiquitously expressed isoform of SNAP25, involved in exocytosis to apical and basolateral domains of epithelial cells [105]. In the cytoplasm SNAP23 is associated with vimentin and thus vimentin filaments are considered a reservoir for SNAP23 that, when needed, is recruited to form SNARE complexes at the plasma membrane [105]. Therefore, vimentin through sequestration of SNAP23 is able to regulate secretion [105].

Other associations between IFs and the vesicular membrane transport machinery of the secretory pathway will be likely discovered in the near future.

### 2.3. Clinical Aspects of the Association between Intermediate Filaments and Vesicular Trafficking in Neurons and Astrocytes

As mentioned above, several links between IFs and the vesicular membrane transport machinery have been discovered in cells of the nervous system [95,101,102,108,109,110]. These findings are of extreme importance in order to understand the molecular mechanisms of the onset of some neurological diseases.

Neurons are the fundamental unit of the nervous system as they receive incoming information and send signals to other cells. Protein synthesis takes place mainly within the cell body of neurons and many proteins must be transported in an anterograde direction to distal regions of the cell. Retrograde transport of proteins from the distal regions to the cell body also occurs. This bidirectional transport has been named axonal transport [130]. In mature neurons, five major IFs are expressed and compose the neuronal IFs: NF-L, NF-M, NF-H, α-internexin and peripherin [30,31,33,131]. Alteration in IF organization affects the axonal transport machinery in DRG (dorsal root ganglion) neurons [109]. Indeed, axonal transport of late endosomes and lysosomes in NF-L-deficient neurons, in neurons overexpressing peripherin or in NF-L deficient neurons overexpressing peripherin was studied observing that neither morphology nor the percentage of moving organelles was affected [109]. In contrast, several kinetic parameters of late endosomal and lysosomal transport, such as the frequency of pauses and of direction changes and the duration of individual movements were altered in NF-L-deficient neurons and in NF-L-deficient neurons overexpressing peripherin [109]. In addition, in neurons lacking NF-L, velocity of late endosomes and lysosomes both in the anterograde and retrograde directions was increased compared to wild type neurons, while in NF-L-deficient neurons overexpressing peripherin only anterograde velocity was incremented [109]. At variance in neurons overexpressing peripherin no major alterations of lysosomal axonal transport were detected [109]. These data indicate that absence of IFs facilitate axonal transport of lysosomes and demonstrate the involvement of IFs in the regulation of fast axonal transport of organelles thus suggesting that alteration in IF dynamics could also contribute to neurodegeneration [109].

Other studies have linked, in a different way, IFs and the endo-lysosomal compartments in neurons as Rab7a was found to interact with peripherin and vimentin, regulating their assembly [95,108]. Interestingly, both proteins have a role in axonal regeneration [25,26] and Rab7a mutant proteins causing the Charcot-Marie-Tooth type 2B peripheral neuropathy seem to interact more strongly with these IF proteins [95,108]. However, a direct role of these vimentin and peripherin in the regulation of the late steps of endocytosis involving Rab7a-positive late endosomal and lysosomal compartments has not yet been demonstrated. Notably, mutations in NF-L have been associated to some forms of Charcot-Marie-Tooth (CMT) disease, causing alteration of NF-L assembly and inhibiting axonal transport of NF-L and mitochondria, thus an effect of NF-L mutations on vesicle transport cannot be excluded in these forms of CMT [132]. Peripherin interacts also with SIP30, a neuronal protein involved in SNAP receptor (SNARE)-dependent exocytosis, suggesting a possible role of peripherin in regulating exocytosis of synaptic vesicles [110].

Therefore, in neurons, IFs are important for the motility of late endosomes and lysosomes between the cell body and the distal regions of the cell, they might regulate exocytosis and they might have a role in vesicular trafficking during axonal regeneration as both vimentin and peripherin are overexpressed in early stages of axonal regeneration [25,26].

An important role of IFs in vesicle motility has been hypothesized also in astrocytes as it has been demonstrated an impairment of vesicle delivery to plasma membrane and of lysosomal motility in astrocytes upon IFs depletion [101,102]. Astrocytes are the most abundant glial cells in the central nervous system (CNS) and actively participate in neuronal functions by communicating with neurons. First of all, astrocytes provide metabolic support to neurons by supplying them with nutrients and removing products of metabolism. Moreover, they regulate neurotransmitter recycling, local ion concentration and pH homeostasis in the extracellular space [133,134,135,136,137,138]. Astrocytes store many neuroactive molecules and some of them are released by vesicles. Calcium is one of the stimuli that mediates the release of a number of neuroactive substances through exocytosis [138,139,140,141,142,143]. Among the substances that are released by exocytosis, there are ATP [144], atrial natriuretic peptide (ANP) [142], glutamate [140] and D-serine [145]. They are transported along the cytoskeleton in vesicles that deliver material to the extracellular space after fusion with the plasma membrane. Subsequently, vesicles may be retrieved back into the cytoplasm [146]. While immature astrocytes contain nestin and vimentin, GFAP become expressed when astrocytes mature. The level of vimentin in mature astrocytes is variable [27,147,148,149,150,151] but, in many neurological disorders, astrocytes undergo several changes showing a reactive phenotype, which is characterized by the increase of vimentin and GFAP expression [37]. Studies conducted in astrocytes lacking GFAP and vimentin demonstrated that vesicle motility is regulated by IFs [101,102]. In fact, the fraction of exocytic ANP-containing vesicles with directional motility was reduced in astrocytes devoid of IFs [101]. Furthermore, after stimulation with both ionomycin and ATP, which increases cytosolic calcium, the motility of endosomes and lysosomes was altered in astrocytes lacking IFs. Indeed, a strong stimulation-dependent reduction in lysosomal motility was observed in wild type astrocytes, but not in astrocytes devoid of IFs [102].

**Figure 1:**
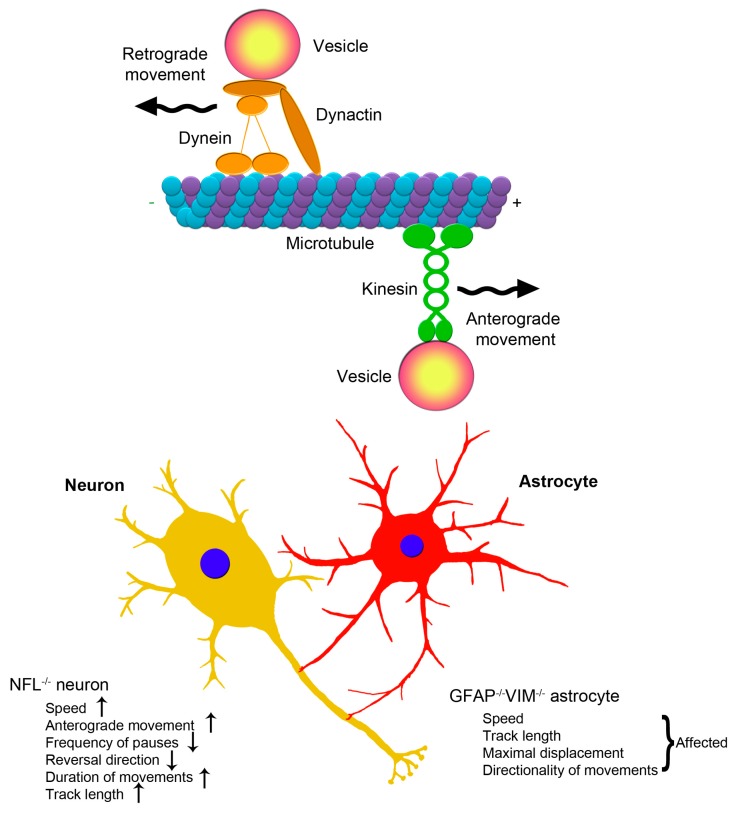
Axonal transport of lysosomes in neurons and astrocytes. Neurons and astrocytes communicate with each other. Both the lack of NFL in neurons and of GFAP and vimentin (VIM) in astrocytes affect lysosome mobility.

In light of the above, IFs play an established important role in endosomal and lysosomal motility both in neurons and in astrocytes [102,109] (Figure 1). Moreover, disruption of IFs affects exocytosis of neuroactive substances in astrocytes and a role of IFs in neuronal exocytosis has also been suggested [101,110] thus altering neuronal functions. Therefore, IFs seem to be necessary for proper brain function by regulating intracellular processes as well as communication between astrocytes and neurons.

As a consequence of the role of IFs in neuronal and astrocytes vesicular trafficking, IFs alterations might be responsible for the onset of several pathologies. The abnormal accumulation of IFs is considered a sign of many neurodegenerative diseases, such as amyotrophic lateral sclerosis (ALS), Parkinson’s disease and Charcot-Marie-Tooth (CMT) disease and several studies proved that disorganization of IFs might be responsible for neuronal dysfunctions [109,132,152,153,154,155,156]. Moreover, it is known that alterations of axonal transport are responsible for the onset of several neurodegenerative disorders [157,158,159,160]. However, the molecular mechanisms underlying these pathologies are still not completely clear.

In light of the emerging data on the involvement of IFs in vesicular trafficking in neurons and astrocytes it can be hypothesized that alterations of IFs responsible for the onset of neuropathies might act by impairing vesicular trafficking. In support of this, studies on axonal transport in case of modulation of IFs, condition which is reminiscent of the IF changes found in ALS, suggest that disorganization of IFs may contribute to neurodegeneration by altering the axonal transport of lysosomes [109,152,161,162,163]. Interestingly, in astrocytes repetitive ionomycin treatment at a short interval induces phosphorylation of vimentin at Ser38 and Ser82 sites, determining a prolongation of phosphorylation at Ser82 and, in turn, disassembly of vimentin filaments [164]. Notably, motility of lysosomes is not decreased by ionomycin treatment in astrocytes lacking vimentin and GFAP whereas it is strongly reduced in control cells [102], thus suggesting that IFs regulates lysosomal motility. A similar mechanism could take place in neurons. Interestingly, Rab7a regulates vimentin phosphorylation state and, in turn, vimentin assembly [95]. CMT2B-associated Rab7a mutants determine higher phosphorylation of vimentin at Ser38 and Ser55 positions than the wild type protein. Moreover, overexpression of CMT2B-causing Rab7a mutants in HeLa cells caused a stronger increase of soluble vimentin compared to the overexpression of wild type Rab7a [95] and similar data were obtained in case of the interaction of Rab7a with peripherin [108]. It will be important to evaluate whether CMT2B-causing Rab7a mutant proteins affect lysosomal motility in axons, in particular during axonal regeneration in peripheral neurons. Further studies on the molecular mechanisms underlying different neuropathies will clarify whether the impairment of the association between IFs and vesicular trafficking might be important for the onset of these diseases.

## 3. Relationship between IFs and Endocytosis during Mitosis

During mitosis, the cell divides to produce two identical daughter cells, which contain the same amount of material shared equally by the mitotic cell during cytokinesis. Phosphorylation of lamins, which are the nuclear IFs, is necessary for the disassembly of the nuclear lamina and for nuclear breakdown at the onset of mitosis [62,63,165].

In vertebrates there are three lamin genes lamin A/C, B1 and B2, from which, by alternative splicing, a number of isoforms are generated. A- and B-type lamins are differentially expressed [166]. For instance, A-type lamins are expressed more abundantly in well-differentiated epithelial cells, whereas B1 lamin is preferentially expressed in proliferating epithelial cells [166].

The main function of lamins is to constitute the karyoskeleton but they have also several other roles such as the regulation of chromatin structure and of signaling pathways playing a crucial role in development [167]. During mitosis, A-type lamins are released into the cytoplasm whereas B-type lamins remain associated with membranes via a lipid post-translational modification [168]. During the telophase/early G1 transition dephosphorylation of lamins is necessary for their assembly and nuclear lamina formation [169]. At variance, cytoplasmic IFs behavior during mitosis is affected by several factors and varies depending on the cell- and IF-type. In fact, during mitosis, in some cell types all IFs are disassembled, in others IF filaments are maintained, whereas in a number of cell types only some kinds of IFs are disassembled [170,171,172].

Studies performed in the 1980s suggested that endocytosis is shut down during mitosis [173,174,175]. Therefore, attention has been focused on the importance of endocytic trafficking in cell division only recently, discovering that endocytosis is fundamental for plasma membrane reshaping during mitosis and for repartition of materials during cytokinesis [176,177]. Interestingly, cyclin-dependent kinase 1 (Cdk1) phosphorylates substrates involved in early endosomes fusion such as RN-tre, a Rab5 GAP, and Vps34 [178,179]. Unphosphorylated Vps34 is responsible for phosphatidylinositol-3-phosphate (PtdIns3P) production on early ensodomes and interacts with beclin [178,179,180]. Early endosomes fusion is regulated by Rab5 and its effector protein early endosomal antigen 1 (EEA1) through the association with PtdIns3P [89,181]. EEA1 is weakly associated with early endosomes during mitosis [182]. Therefore, Cdk1 might be responsible for the inhibition of endosomes fusion mediated by RN-tre, Vps34 and EEA1. Other proteins of the vesicular transport machinery have been associated with cell division. In fact, it has been demonstrated that the GTPases Arf6, Rab4, Rab11 and ESCRT (Endosomal Sorting Complex Required for Transport) proteins are localized either in the proximity of the spindle midzone or at the cytokinetic furrow and the midbody [183,184,185,186]. Moreover, impairment of the GTPase Rab6A′ function, which regulates a retrograde transport route connecting early endosomes and the endoplasmic reticulum in interphase, leads to a block in metaphase [187]. In addition, it has been demonstrated that Rab35 is required for cytokinesis [188]. All these data strongly suggest a connection between endocytosis and mitosis.

The first link between vesicular trafficking and IFs during mitosis has been proposed when it was demonstrated that Rab5 depletion delays the disassembly of the nuclear lamina in *Caenorhabditis* *elegans* [189]. Then a role for Rab5 in chromosome alignment during Drosophila mitosis through the regulation of Lamin disassembly and of localization of Mud, two components of the interphase nuclear envelope, was proposed [112]. Lamin is the single Drosophila homolog of the two vertebrate B-type lamins. The nuclear envelope in *Drosophila* and *C. elegans* embryos, unlike in vertebrates, is only partially disrupted at spindle poles and, as nuclear pore complexes disassemble, it generates a fenestrated nuclear envelope present in many Drosophila tissues and called spindle envelope. Rab5 localizes around the spindle poles and it associates with vesicles that move to centrosomes along astral microtubules in a dynein-dependent manner. Moreover, Rab5 forms a complex *in vivo* with Lamin and Mud, and Rab5 depletion causes an increase of prometaphase/metaphase cells showing Lamin around the spindle envelope affecting also the localization at poles of Mud and other factors. Thus, it has been suggested that Rab5, regulates proper disassembly of the nuclear lamina in mitosis and, through the interaction with Lamin and Mud, influences spindle orientation, kinetochore tension and chromosome alignment [112].

A stronger connection between vimentin and endocytosis during mitosis was demonstrated recently in HeLa cells where vimentin remains filamentous during mitosis and associates with early endosomes in M-phase-arrested cells [97]. In fact, it was established that vimentin phosphorylation on Ser459 is mediated by Polo-like kinase (Plk) 1, does not affect vimentin polymerization but regulates Rab21 association with β1-integrin vesicles during metaphase [97]. Moreover, vimentin Ser459 phosphorylation is required for the Rab21-regulated β1-integrin localization at the cleavage furrow during telophase and, in turn, for cytokinesis [97,190]. In addition, Plk1 regulates early endosomes fusion during mitosis as after Plk1 or vimentin depletion early endosomes, but not late endosomes, are enlarged in M-phase due to an abnormal endocytic vescicle fusion that depends on vimentin phosphorylation state [97]. As early endosome fusion depends on the activity of Rab5 and EEA1, it will be interesting to investigate in more details the role of Plk1 and vimentin on these proteins. It was also demonstrated that Plk1 depletion does not influence Rab5 activity or association with β1-integrin vesicles although an effect on Rab5 localization during mitosis cannot be excluded considering that Rab5 has been shown to interact with vimentin and to be required for chromosome alignment and that vimentin is associated with early endosomes [97,100,112]. In this process, it would be interesting to evaluate the role of Rab7a, as Rab7a has been found in endosome fractionations prepared from M phase arrested cells and interacts with vimentin [95,97]. Moreover, it would be also interesting to investigate whether Rab7a, which modulates vimentin phoshorylation state [95], is able to affect vimentin phosphorylation on Ser459 site.

**Figure 2:**
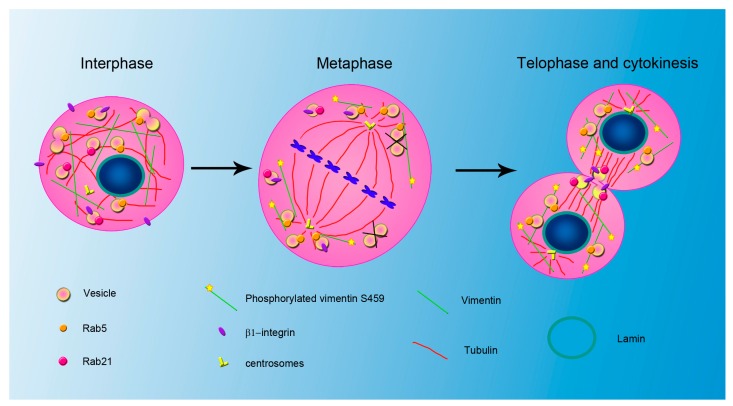
IFs and vesicle trafficking during cell cycle. Model representing the suggested role of Rab5, Rab21, vimentin filaments and lamins during interphase and mitosis. In interphase, Rab5-positive vesicles can localize also around the nucleus and fusion between these organelles occurs. In metaphase, nuclear lamina disassembly is controlled also by Rab5 and Rab5-positive organelles localize around the spindle poles. At this stage, vimentin filaments are phosphorylated on Ser459 and regulate Rab21 but not Rab5 association with β1-integrin-containing vesicles. Thus, vimentin is associated with endosomes, whose fusion is blocked in metaphase. At later stages lamins are dephosphorylated, the nuclear lamina is assembled and vimentin phosphorylation on Ser459 is required for Rab21-positive β1-integrin vesicles localization at the cleavage furrow where they fuse contributing to separation of daughter cells. Rab5-positive vesicles accumulate around the newly reformed nuclear envelope.

The data collected on the associations Rab5/lamins and vimentin/Rab21/early endosomes (Figure 2) prove a direct and strong connection between IFs and vesicular trafficking in mitosis whose role was previously underestimated. In particular, these data demonstrate that the relationship between endocytosis and IFs might be important for several stages and mechanisms of mitosis. New links between IFs and endosomal trafficking will probably be established in the future, increasing the knowledge of the molecular events occurring during mitosis and involving vesicular traffic.

## 4. Conclusions

It is now clear that IFs are not just a static cellular component and that cells rely on IF functions for many important processes. Indeed, IF functions include cell mechanical support, organization of the other cytoskeleton components, regulation of nucleus structure and activity, cell signaling, cell migration, cell growth, survival and apoptosis. In recent years, the interest in the relationship between IFs and vesicular trafficking has increased, due to the discovery of several key interactions between IFs and components of the molecular machinery regulating vesicular trafficking. Further studies are certainly required to clarify the functional meaning of these interactions but, nevertheless, a consistent amount of data indicate the involvement of IFs in the regulation of a number of steps of vesicular trafficking both in interphase and mitosis.

In particular, interactions between IFs and key proteins of sorting and recycling early endosomes have been established and, functional relationships between early stages of endocytosis and IFs have been detected during mitosis. Further studies on the role of IFs in the first steps of endocytosis, whose function in mitosis has long been underestimated, will be of importance to clarify the molecular mechanism of a number of events occurring during cell division. Other evidences support the importance of IFs in the late stages of endocytosis. In fact, it has been established that IFs interact with key proteins of the late endocytic trafficking, being important for the regulation of the luminal content of late endosomes and lysosomes, but also for their distribution and motility. Moreover, IFs seem to affect also autophagy, which overlap with endocytosis, as both share lysosomal degradation as the common terminal end-point. As lysosomal degradation plays a key role in several diseases and alterations of IFs have been associated to a number of neuropathies, the link between IFs and vesicular trafficking might explain the pathological mechanism and might be determinant in the onset of these pathologies. Furthermore, the association between IFs and exocytosis might be relevant in the regulation of neurotransmitters release and therefore in brain physiological functions and pathology.

Understanding in details IFs functions in vesicular trafficking will surely open new scenario not only on different aspects of cell biology but also on the molecular mechanisms underlying a number of diseases.

## Figures and Tables

**Table 1:** List of links between IFs and the vesicular membrane transport machinery.

IF	Interaction	Function in Vesicular Trafficking
Keratin 8		Formation of autophagosomes [93]
		Syntaxin 3 targeting [94]
Vimentin	Rab7a regulates vimentin phosphorylation state and assembly [95]	
		Endocytosis of Jagged-1 [96]
		Inhibition of endocytic vesicles fusion in mitosis [97]
		Rab21-regulated β1-integrin trafficking to the cleavage furrow [97]
	AP-3 [98]	Positioning of late endosomal-lysosomal compartments, luminal ionic composition of endocytic organelles and content of autophagosomes [98]
	Rab9 [99]	
	Rab5 [100]	
		Directional mobility of vesicles [101]
		Activity-dependent mobility of endosomes/lysosomes [102,103]
		Integrin recycling [104]
		Reservoir for SNAP23 [105]
	Rab4A [106]	
		Intracellular transport of glicolipids [107]
GFAP		Directional mobility of vesicles [101]
		Endocytosis of Jagged-1 [96]
		Activity-dependent mobility of endosomes/lysosomes [102,103]
Peripherin	Rab7a regulates peripherin assembly [108]	
	AP-3 [98]	
		Lysosomal transport [109]
	SIP30 affects peripherin assembly [110]	Subcellular distribution of SIP30 and SNAP25 [110]
Desmin		Lysosomal distribution [111]
	Rab5 [100]	
NF-L		Lysosomal transport [109]
α-Internexin	AP-3 [98]	
Drosophila lamin	Rab5 regulates lamin disassembly [112]	

## Abbreviations

The following abbreviations are used in this manuscript:

ALS	amyotrophic lateral sclerosis
ANP	atrial natriuretic peptide
BLOC-1	biogenesis of lysosome-related organelles complex 1
Cdk1	cyclin-dependent kinase 1
CMT	Charcot-Marie-Tooth
CMT2B	Charcot-Marie-Tooth type 2B
CNS	central nervous system
DRG	dorsal root ganglion
ERK	extracellular-signal-regulated kinase
GCP6	γ-tubulin complex protein 6
GFAP	glial fibrillary acidic protein
IFs	intermediate filament proteins
LAMP-1	Lysosomal-associated membrane protein 1
LAMP-2	Lysosomal-associated membrane protein 2
MDC	Monodansylcadaverine
NF	Neurofilament
NF-H	neurofilament heavy
NF-L	neurofilament light
NF-M	neurofilament medium
NPC1	Niemann-Pick type C1
Plk1	Polo-like kinase 1
PtdIns3P	phosphatidylinositol-3-phosphate
SNARE	SNAP (Soluble NSF Attachment Protein) REceptor

## References

[1] Hesse M., Magin T.M., Weber K. (2001). Genes for intermediate filament proteins and the draft sequence of the human genome: Novel keratin genes and a surprisingly high number of pseudogenes related to keratin genes 8 and 18. J. Cell Sci..

[2] Rogers M.A., Edler L., Winter H., Langbein L., Beckmann I., Schweizer J. (2005). Characterization of new members of the human type II keratin gene family and a general evaluation of the keratin gene domain on chromosome 12q13.13. J. Investig. Dermatol..

[3] Rogers M.A., Winter H., Langbein L., Bleiler R., Schweizer J. (2004). The human type I keratin gene family: Characterization of new hair follicle specific members and evaluation of the chromosome 17q21.2 gene domain. Differentiation.

[4] Szeverenyi I., Cassidy A.J., Chung C.W., Lee B.T., Common J.E., Ogg S.C., Chen H., Sim S.Y., Goh W.L., Ng K.W. (2008). The human intermediate filament database: Comprehensive information on a gene family involved in many human diseases. Hum. Mutat..

[5] Ishikawa H., Bischoff R., Holtzer H. (1968). Mitosis and intermediate-sized filaments in developing skeletal muscle. J. Cell Biol..

[6] Goldman R.D., Follett E.A. (1969). The structure of the major cell processes of isolated BHK21 fibroblasts. Exp. Cell. Res..

[7] Goldman R.D., Follett E.A. (1970). Birefringent filamentous organelle in BHK-21 cells and its possible role in cell spreading and motility. Science.

[8] Franke W.W., Schmid E., Osborn M., Weber K. (1978). Different intermediate-sized filaments distinguished by immunofluorescence microscopy. Proc. Natl. Acad. Sci. USA.

[9] Geisler N., Weber K. (1981). Comparison of the proteins of two immunologically distinct intermediate-sized filaments by amino acid sequence analysis: Desmin and vimentin. Proc. Natl. Acad. Sci. USA.

[10] Fuchs E., Weber K. (1994). Intermediate filaments: Structure, dynamics, function, and disease. Annu. Rev. Biochem..

[11] Herrmann H., Strelkov S.V., Burkhard P., Aebi U. (2009). Intermediate filaments: Primary determinants of cell architecture and plasticity. J. Clin. Investig..

[12] Parry D.A., Strelkov S.V., Burkard P., Aebi U., Herrmann H. (2007). Towards a molecular description of intermediate filament structure and assembly. Exp. Cell Res..

[13] Geisler N., Weber K. (1982). The amino acid sequence of chicken muscle desmin provides a common structural model for intermediate filaments proteins. EMBO J..

[14] Quax-Jeuken Y.E., Quax W.J., Bloemendal H. (1983). Primary and secondary structure of hamster vimentin predicted from the nucleotide sequence. Proc. Natl. Acad. Sci. USA.

[15] Strelkov S.V., Hermann H., Aebi U. (2003). Molecular architecture of intermediate filaments. Bioessays.

[16] Izawa I., Inagaki M. (2006). Regulatory mechanisms and functions of intermediate filaments: A study using site- and phosphorylation state-specific antibodies. Cancer Sci..

[17] Kornreich M., Avinery R., Malka-Gibor E., Laser-Azogui A., Beck R. (2015). Order and disorder in intermediate filament proteins. FEBS Lett..

[18] Miller R.K., Khuon S., Goldman R.D. (1993). Dynamics of keratin assembly: Exogenous type I keratin rapidly associates with type II keratin *in vivo*. J. Cell Biol..

[19] Quinlan R.A., Franke W.W. (1982). Heteropolymer filaments of vimentin and desmin in vascular smooth muscle tissue and cultured Baby Hamster Kidney cells demonstrated by chemical crosslinking. Proc. Natl. Acad. Sci. USA.

[20] Quinlan R.A., Franke W.W. (1983). Molecular interactions in intermediate-sized filaments revealed by chemical cross-linking. Heteropolymers of vimentin and glial filament protein in cultured human glioma cells. Eur. J. Biochem..

[21] Monteiro M.J., Cleveland D.W. (1989). Expression of NF-L and NF-M in fibroblasts reveals coassembly of neurofilament and vimentin subunits. J. Cell Biol..

[22] Lilienbaum A., Legagneux V., Portier M.M., Dellagi K., Paulin D. (1986). Vimentin gene: Expression in human lymphocytes and in Burkitt’s lymphoma cells. EMBO J..

[23] Franke W.W., Schmid E., Osborn M., Weber K. (1979). Intermediate-sized filaments of human endothelial cells. J. Cell Biol..

[24] Schmid E., Tapscott S., Bennett G.S., Croop J., Fellini S.A., Holtzer H., Franke W.W. (1979). Differential location of different types of intermediate-sized filaments in various tissues of the chicken embryo. Differentiation.

[25] Shea T.B., Beermann M.L., Fischer I. (1993). Transient requirement for vimentin in neuritogenesis: Intracellular delivery of anti-vimentin antibodies and antisense oligonucleotides inhibit neurite initiation but not elongation of existing neurites in neuroblastoma. J. Neurosci. Res..

[26] Cochard P., Paulin D. (1984). Initial expression of neurofilaments and vimentin in the central and peripheral nervous system of the mouse embryo *in vivo*. J. Neurosci..

[27] Lazarides E. (1982). Intermediate filaments: A chemically heterogeneous, developmentally regulated class of proteins. Annu. Rev. Biochem..

[28] Chiu F.C., Norton W.T., Fields K.L. (1981). The cytoskeleton of primary astrocytes in culture contains actin, glial fibrillary acidic protein, and the fibroblast-type filament protein, vimentin. J. Neurochem..

[29] Jessen K.R., Mirsky R. (1980). Glial cells in the enteric nervous system contain glial fibrillary acidic protein. Nature.

[30] Portier M.M., de Néchaud B., Gros F. (1984). Peripherin, a new member of the intermediate filament protein family. Dev. Neurosci..

[31] Portier M.M., Escurat M., Landon F., Djabali K., Bousquet O. (1993). Peripherin and neurofilaments: Expression and role during neural development. C. R. Acad. Sci. III.

[32] Errante L., Tang D., Gardon M., Sekerkova G., Mugnaini E., Shaw G. (1998). The intermediate filament protein peripherin is a marker for cerebellar climbing fibres. J. Neurocytol..

[33] Pannese E. (1962). Detection of neurofilaments in the perikaryon of hypertrophic nerve cells. J. Cell Biol..

[34] Toivola D.M., Boor P., Alam C., Strnad P. (2015). Keratins in health and disease. Curr. Opin. Cell Biol..

[35] Norgren N., Rosengren L., Stigbrand T. (2003). Elevated neurofilament levels in neurological diseases. Brain Res..

[36] Gaiottino J., Norgren N., Dobson R., Topping J., Nissim A., Malaspina A., Bestwick J.P., Monsch A.U., Regeniter A., Lindberg R.L. (2013). Increased neurofilament light chain blood levels in neurodegenerative neurological diseases. PLoS ONE.

[37] Hol E.M., Pekny M. (2015). Glial fibrillary acidic protein (GFAP) and the astrocyte intermediate filament system in diseases of the central nervous system. Curr. Opin. Cell Biol..

[38] Xiao S., McLean J., Robertson J. (2006). Neuronal intermediate filaments and als: A new look at an old question. Biochim. Biophys. Acta.

[39] Wang N., Stamenovic D. (2000). Contribution of intermediate filaments to cell stiffness, stiffening, and growth. Am. J. Physiol..

[40] Haudenschild D., Chen J., Pang N., Steklov N., Grogan S.P., Lotz M.K., D’Lima D.D. (2011). Vimentin contributes to changes in chondrocyte stiffness in osteoarthritis. J. Orthop. Res..

[41] Guo M., Ehrlicher A.J., Mahammad S., Fabich H., Jensen M.H., Moore J.R., Fredberg J.J., Goldman R.D., Weitz D.A. (2013). The role of vimentin intermediate filaments in cortical and cytoplasmic mechanics. Biophys. J..

[42] Mendez M.G., Restle D., Janmey P.A. (2014). Vimentin enhances cell elastic behaviour and protects against compressive stress. Biophys. J..

[43] Bordeleau F., Myrand Lapierre M.-E., Sheng Y., Marceau N. (2012). Keratin 8/18 regulation of cell stiffness-extracellular matrix interplay through modulation of rho-mediated actin cytoskeleton dynamics. PLoS ONE.

[44] Hutchison C.J. (2002). Lamins: Building blocks or regulators of gene expression?. Nat. Rev. Mol. Cell Biol..

[45] Bloom S., Lockard V.G., Bloom M. (1996). Intermediate filament-mediated stretch-induced changes in chromatin: A hypothesis for growth initiation in cardiac myocytes. J. Mol. Cell. Cardiol..

[46] Tolstonog G.V., Sabasch M., Traub P. (2002). Cytoplasmatic intermediate filaments are stably associated with nuclear matrices and potentially modulate their DNA-binding function. DNA Cell Biol..

[47] Kiseleva E., Allen T.D., Rutherford S., Bucci M., Wente S.R., Goldberg M.W. (2004). Yeast nuclear pore complexes have a cytoplasmic ring and internal filaments. J. Struct. Biol..

[48] Stoffler D., Feja B., Fahrenkrog B., Walz J., Typke D., Aebi U. (2003). Cryo-electron tomography provides novel insights into nuclear pore architecture: Implications for nucleocytoplasmic transport. J. Mol. Biol..

[49] Ellis D.J., Jenkins H., Whitfield W.G., Hutchinson C.J. (1997). GST-lamin fusion proteins act as dominant negative mutants in xenopus egg extract and reveal the function of the lamina in DNA replication. J. Cell Sci..

[50] Spann T.P., Moir R.D., Goldman A.E., Stick R., Goldman R.D. (1997). Disruption of nuclear lamin organization alters the distribution of replication factors and inhibits DNA synthesis. J. Cell Biol..

[51] Solovei I., Wang A.S., Thanisch K., Schmidt C.S., Krebs S., Zwerger M., Cohen T.V., Devys D., Foisner R., Peichl L. (2013). LBR and lamina A/C sequentially tether peripheral heterochromatin and inversely regulate differentiation. Cell.

[52] Chaturvedi P., Parnaik V.K. (2010). Lamin A rod domain mutants target heterochromatin protein 1alpha and beta for proteasomal degradation by activation of F-box protein, fbxw10. PLoS ONE.

[53] Zhang S., Schones D.E., Malicet C., Rochman M., Zhou M., Foisner R., Bustin M. (2013). High mobility group protein N5 (HMGN5) and lamina-associated polypeptide 2α (LAP2α) interact and reciprocally affect their genome-wide chromatin organization. J. Biol. Chem..

[54] Rochman M., Malicet C., Bustin M. (2010). Hmgn5/nsbp1: A new member of the HMGN protein family that affects chromatin structure and function. Biochim. Biophys. Acta.

[55] Montes de Oca R., Andreassen P.R., Wilson K.L. (2011). Barrier-to-autointegration factor influences specific histone modifications. Nucleus.

[56] Spann T.P., Goldman A.E., Wang C., Huang S., Goldman R.D. (2002). Alteration of nuclear lamin organization inhibits rna polymerase ii-dependent transcription. J. Cell Biol..

[57] Manjiu K., Muralikrishna B., Parnaik V.K. (2006). Expression of disease-causing lamin A mutants impairs the formation of DNA repair foci. J. Cell Sci..

[58] Gibbs-Seymour I., Markiewicz E., Bekker-Jensen S., Mailand N., Hutchison C.J. (2015). Lamin A/C-dependent interaction with 53BP1 promotes cellular responses to DNA damage. Aging Cell.

[59] Ghosh S., Liu B., Wang Y., Hao Q., Zhou Z. (2015). Lamin a is an endogenous SIRT6 activator and promotes SIRT6-mediated DNA repair. Cell Rep..

[60] Gonzalez-Suarez I., Redwood A.B., Perkins S.M., Vermolen B., Lichtensztejin D., Grotsky D.A., Morgado-Palacin L., Gapud E.J., Sleckman B.P., Sullivan T. (2009). Novel roles for A-type lamins in telomere biology and the DNA damage response pathway. EMBO J..

[61] Beaudoin J., Gerlich D., Daigle N., Eils R., Ellenberg J. (2002). Nuclear envelope breakdown proceeds by microtubule-induced tearing of the lamina. Cell.

[62] Ward G.E., Kirschner M.W. (1990). Identification of cell-cycle regulated phosphorylation sites on nuclear lamin C. Cell.

[63] Peter M., Nakagawa J., Dorée M., Labbé J.C., Nigg E.A. (1990). *In vitro* disassembly of the nuclear lamina and M phase-specific phosphorylation of lamins by cdc2 kinase. Cell.

[64] Kochin V., Shimi T., Torvaldson E., Adam S.A., Goldman A., Pack C.G., Melo-Cardenas J., Imanishi S.Y., Goldman R.D., Eriksson J.E. (2014). Interphase phosphorylation of lamin A. J. Cell Sci..

[65] Coffinier C., Jung H.J., Nobumori C., Chang S., Tu Y., Barnes R.H.N., Yoshinaga Y., de Jong P.J., Vergnes L., Reue K. (2011). Deficiencies in lamin B1 and lamin B2 cause neurodevelopmental defects and distinct nuclear shape abnormalities in neurons. Mol. Biol. Cell.

[66] Sarria A.J., Lieber J.G., Nordeen S.K., Evans R.M. (1994). The presence or absence of a vimentin-type intermediate filament network affects the shape of the nucleus in human SW-13 cells. J. Cell Sci..

[67] Oriolo A.S., Wald F.A., Canessa G., Salas P.J. (2006). GCP6 binds to intermediate filaments: A novel function of keratins in the organization of microtubules in epithelial cells. Mol. Biol. Cell.

[68] Conover G.M., Gregorio C.C. (2011). The desmin coil 1b mutation K190A impairs nebulin Z-disc assembly and destabilizes actin thin filaments. J. Cell Sci..

[69] Cary R.B., Klymkowsky M.W., Evans R.M., Domingo A., Dent J.A., Backhus L.E. (1994). Vimentin’s tail interacts with actin-containing structures *in vivo*. J. Cell Sci..

[70] Esue O., Carson A.A., Tseng Y., Wirtz D. (2006). A direct interaction between actin and vimentin filaments mediated by the tail domain of vimentin. J. Biol. Chem..

[71] Virtakoivu R., Mai A., Mattila E., De Franceschi N., Imanishi S.Y., Corthals G., Kaukonen R., Saari M., Cheng F., Torvaldson E. (2015). Vimentin-ERK signaling uncouples Slug gene regulatory function. Cancer Res..

[72] Perlson E., Hanz S., Ben-Yaakov K., Segal-Ruder Y., Seger R., Fainzilber M. (2005). Vimentin-dependent spatial translocation of an activated MAP kinase in injured nerve. Neuron.

[73] Perlson E., Michaelevski I., Kowalsman N., Ben-Yaakov K., Shaked M., Seger R., Eisenstein M., Fainzilber M. (2006). Vimentin binding to phosphorylated ERK sterically hinders enzymatic dephosphorylation of the kinase. J. Mol. Biol..

[74] Kumar N., Robidoux J., Daniel K.W., Guzman G., Floering L.M., Collins S. (2007). Requirement of vimentin filament assembly for beta3-adrenergic receptor activation of ERK MAP kinase and lipolysis. J. Biol. Chem..

[75] Toivola D.M., Nieminen M.I., Hesse M., He T., Baribault H., Magin T.M., Omary M.B., Eriksson J.E. (2001). Disturbances in hepatic cell-cycle regulation in mice with assembly-deficient keratins 8/18. Hepatology.

[76] Caulin C., Ware C.F., Magin T.M., Oshima R.G. (2000). Keratin-dependent, epithelial resistance to tumor necrosis factor-induced apoptosis. J. Cell Biol..

[77] Rao L., Perez D., White E. (1996). Lamin proteolysis facilitates nuclear events during apoptosis. J. Cell Biol..

[78] Gilles C., Polette M., Zahm J.M., Turnier J.M., Volders L., Foidart J.M., Birembaut P. (1999). Vimentin contributes to human mammary epithelial cell migration. J. Cell Sci..

[79] Sutoh Yoneyama M., Hatakeyama S., Habuchi T., Inoue T., Nakamura T., Funyu T., Wiche G., Ohyama C., Tsuboi S. (2014). Vimentin intermediate filament and plectin provide a scaffold for invadopodia, facilitating cancer cell invasion and extravasation for metastasis. Eur. J. Cell Biol..

[80] Ju J.H., Yang W., Lee K.M., Oh S., Nam K., Shim S., Shin S.Y., Gye M.C., Chu I.S., Shin I. (2013). Regulation of cell proliferation and migration by keratin 19-induced nuclear import of early growth response-1 in breast cancer cells. Clin. Cancer Res..

[81] Fortier A.M., Asselin E., Cadrin M. (2013). Keratin 8 and 18 loss in epithelial cancer cells increases collective cell migration and cisplatin sensitivity through claudin1 up-regulation. J. Biol. Chem..

[82] Narita K., Matsuda Y., Seike M., Naito Z., Gemma A., Ishiwata T. (2014). Nestin regulates proliferation, migration, invasion and stemness of lung adenocarcinoma. Int. J. Oncol..

[83] Jahn R. (2004). Principles of exocytosis and membrane fusion. Ann. N. Y. Acad. Sci..

[84] Brooks D.A. (2009). The endosomal network. Int. J. Clin. Pharmacol. Ther..

[85] Bhuin T., Roy J.K. (2014). Rab proteins: The key regulators of intracellular vesicle transport. Exp. Cell Res..

[86] Bucci C., Parton R.G., Mather I.H., Stunnenberg H., Simons K., Hoflack B., Zerial M. (1992). The small GTPase Rab5 functions as a regulatory factor in the early endocytic pathway. Cell.

[87] Nielsen E., Severin F., Backer J.M., Hyman A.A., Zerial M. (1999). Rab5 regulates motility of early endosomes on microtubules. Nat. Cell Biol..

[88] Zeigerer A., Gilleron J., Bogorad R.L., Marsico G., Nonaka H., Seifert S., Epstein-Barash H., Kuchimanchi S., Peng C.G., Ruda V.M. (2012). Rab5 is necessary for the biogenesis of the endolysosomal system *in vivo*. Nature.

[89] Gorvel J.P., Chavrier P., Zerial M., Gruenberg J. (1991). Rab5 controls early endosome fusion *in vitro*. Cell.

[90] McCaffrey M.W., Bielli A., Cantalupo G., Mora S., Roberti V., Santillo M., Drummond F., Bucci C. (2001). Rab4 affects both recycling and degradative endosomal trafficking. FEBS Lett..

[91] Ullrich O., Reinsch S., Urbé S., Zerial M., Parton R.G. (1996). Rab11 regulates recycling through the pericentriolar recycling endosome. J. Cell Biol..

[92] Casanova J.E., Wang X., Kumar R., Bhartur S.G., Navarre J., Woodrum J.E., Altschuler Y., Ray G.S., Goldenring J.R. (1999). Association of Rab25 and Rab11a with the apical recycling system of polarized Madin-Darby canine kidney cells. Mol. Biol. Cell.

[93] Blankson H., Holen I., Seglen P.O. (1995). Disruption of the cytokeratin cytoskeleton and inhibition of hepatocytic autophagy by okadaic acid. Exp. Cell Res..

[94] Ameen N.A., Figueroa Y., Salas P.J. (2001). Anomalous apical plasma membrane phenotype in CK8-deficient mice indicates a novel role for intermediate filaments in the polarization of simple epithelia. J. Cell Sci..

[95] Cogli L., Progida C., Bramato R., Bucci C. (2013). Vimentin phosphorylation and assembly are regulated by the small GTPase Rb7a. Biochim. Biophys. Acta.

[96] Wilhelmsson U., Faiz M., de Pablo Y., Sjöqvist M., Andersson D., Widestrand A., Potokar M., Stenovec M., Smith P.L., Shinjyo N. (2012). Astrocytes negatively regulate neurogenesis through the Jagged1-mediated Notch pathway. Stem Cells.

[97] Ikawa K., Satou A., Fukuhara M., Matsumura S., Sugiyama N., Goto H., Fukuda M., Inagaki M., Ishihama Y., Toyoshima F. (2014). Inhibition of endocytic vesicle fusion by Plk1-mediated phosphorylation of vimentin during mitosis. Cell Cycle.

[98] Styers M.L., Salazar G., Love R., Peden A.A., Kowalczyk A.P., Faundez V. (2004). The endo-lysosomal sorting machinery interacts with the intermediate filament cytoskeleton. Mol. Biol. Cell.

[99] Walter M., Chen F.W., Tamari F., Wang R., Ioannou Y.A. (2009). Endosomal lipid accumulation in NPC1 leads to inhibition of PKC, hypophosphorylation of vimentin and Rab9 entrapment. Biol. Cell.

[100] Kurzchalia T.V., Gorvel J.P., Dupree P., Parton R., Kellner R., Houthaeve T., Gruenberg J., Simons K. (1992). Interactions of Rab5 with cytosolic proteins. J. Biol. Chem..

[101] Potokar M., Kreft M., Li L., Daniel Andersson J., Pangrsic T., Chowdhury H.H., Pekny M., Zorec R. (2007). Cytoskeleton and vesicle mobility in astrocytes. Traffic.

[102] Potokar M., Stenovec M., Gabrijel M., Li L., Kreft M., Grilc S., Pekny M., Zorec R. (2010). Intermediate filaments attenuate stimulation-dependent mobility of endosomes/lysosomes in astrocytes. Glia.

[103] Vardjan N., Gabrijel M., Potokar M., Svajger U., Kreft M., Jeras M., de Pablo Y., Faiz M., Pekny M., Zorec R. (2012). IFN-γ-induced increase in the mobility of MHC class II compartments in astrocytes depends on intermediate filaments. J. Neuroinflamm..

[104] Ivaska J., Vuoriluoto K., Huovinen T., Izawa I., Inagaki M., Parker P.J. (2005). PKCepsilon-mediated phosphorylation of vimentin controls integrin recycling and motility. EMBO J..

[105] Faigle W., Colucci-Guyon E., Louvard D., Amigorena S., Galli T. (2000). Vimentin filaments in fibroblasts are a reservoir for SNAP23, a component of the membrane fusion machinery. Mol. Biol. Cell.

[106] Mruk D.D., Lau A.S., Sarkar O., Xia W. (2007). Rab4a GTPase catenin interactions are involved in cell junction dynamics in the testis. J. Androl..

[107] Gillard B.K., Clement R., Colucci-Guvon E., Babinet C., Schwarzmann G., Taki T., Kasama T., Marcus D.M. (1998). Decreased synthesis of glycosphingolipids in cells lacking vimentin intermediate filaments. Exp. Cell Res..

[108] Cogli L., Progida C., Thomas C.L., Spencer-Dene B., Donno C., Schiavo G., Bucci C. (2013). Charcot-Marie-Tooth type 2b disease-causing Rab7a mutant proteins show altered interaction with the neuronal intermediate filament peripherin. Acta Neuropathol..

[109] Perrot R., Julien J.P. (2009). Real-time imaging reveals defects of fast axonal transport induced by disorganization of intermediate filaments. FASEB J..

[110] Gentil B.J., McLean J.R., Xiao S., Zhao B., Durham H.D., Robertson J. (2014). A two-hybrid screen identifies an unconventional role for the intermediate filament peripherin in regulating the subcellular distribution of the SNAP25-interacting protein, SIP30. J. Neurochem..

[111] Kouloumenta A., Mayroidis M., Capetanaki Y. (2007). Proper perinuclear localization of the TRIM-like protein myospryn requires its binding partner desmin. J. Biol. Chem..

[112] Capalbo L., D’Avino P.P., Archambault V., Glover D.M. (2011). Rab5 GTPase controls chromosome alignment through Lamin disassembly and relocation of the NuMA-like protein Mud to the poles during mitosis. Proc. Natl. Acad. Sci. USA.

[113] Ivaska J., Whelan R.D., Watson R., Parker P.J. (2002). PKC epsilon controls the traffic of beta1 integrins in motile cells. EMBO J..

[114] Bucci C., Frunzio R., Chiariotti L., Brown A.L., Rechler M.M., Bruni C.B. (1988). A new member of the ras gene superfamily identified in a rat liver cell line. Nucleic Acids Res..

[115] Bucci C., Thomsen P., Nicoziani P., McCarthy J., van Deurs B. (2000). Rab7: A key to lysosome biogenesis. Mol. Biol. Cell.

[116] Harrison R., Bucci C., Vieira O., Schroer T., Grinstein S. (2003). Phagosomes fuse with late endosomes and/or lysosomes by extension of membrane protrusions along microtubules: Role of Rab7 and RILP. Mol. Cell. Biol..

[117] Jager S., Bucci C., Tanida I., Ueno T., Kominami E., Saftig P., Eskelinen E.L. (2004). Role for Rab7 in maturation of late autophagic vacuoles. J. Cell Sci..

[118] Riederer M.A., Soldati T., Shapiro A.D., Lin J., Pfeffer S.R. (1994). Lysosome biogenesis requires Rab9 function and receptor recycling from endosomes to the trans-golgi network. J. Cell Biol..

[119] Ganley I.G., Carroll K., Bittova L., Pfeffer S. (2004). Rab9 GTPase regulates late endosome size and requires effector interaction for its stability. Mol. Biol. Cell.

[120] Ooi C.E., Dell’Angelica E.C., Bonifacino J.S. (1998). ADP-Ribosylation Factor 1 (ARF1) regulates recrutiment of the AP-3 adaptor complex to membranes. J. Cell Biol..

[121] Drake M.T., Zhu Y., Kornfeld S. (2000). The assembly of AP-3 adaptor complex-containing clathrin-coated vesicles on synthetic liposomes. Mol. Biol. Cell.

[122] Styers M.L., Kowalczyk A.P., Faundez V. (2006). Architecture of the vimentin cytoskeleton is modified by perturbation of the GTPase ARF1. J. Cell Sci..

[123] Mihai C., Chrisler W.B., Xie Y., Hu D., Szymanski C.J., Tolic A., Klein J.A., Smith J.N., Tarasevich B.J., Orr G. (2015). Intracellular accumulation dynamics and fate of zinc ions in alveolar epithelial cells exposed to airborne ZNO nanoparticles at the air-liquid interface. Nanotoxicology.

[124] Kobayashi T., Beuchat M.H., Lindsay M., Frias S., Palmiter R.D., Sakuraba H., Parton R.G., Gruenberg J. (1999). Late endosomal membranes rich in lysobisphosphatidic acid regulate cholesterol transport. Nat. Cell Biol..

[125] Salazar G., Love R., Styers M.L., Werner E., Peden A., Rodriguez S., Gearing M., Wainer B.H., Faundez V. (2004). AP-3-dependent mechanisms control the targeting of a chloride channel (ClC-3) in neuronal and non-neuronal cells. J. Biol. Chem..

[126] Holen I., Gordon P.B., Seglen P.O. (1992). Protein kinase-dependent effects of okadaic acid on hepatocytic autophagy and cytoskeletal integrity. Biochem. J..

[127] Milner D.J., Mayroidis M., Weisleder N., Capetanaki Y. (2000). Desmin cytoskeleton linked to muscle mitochondrial distribution and respiratory function. J. Cell Biol..

[128] Eskelinen E.L., Illert A.L., Tanaka Y., Schwarzmann G., Blanz J., Von Figura K., Saftig P. (2002). Role of LAMP-2 in lysosome biogenesis and autophagy. Mol. Biol. Cell.

[129] Salas P.J., Rodriguez M.L., Viciana A.L., Vega-Salas D.E., Hauri H.P. (1997). The apical submembrane cytoskeleton participates in the organization of the apical pole in epithelial cells. J. Cell Biol..

[130] Grafstein B., Forman D.S. (1980). Intracellular transport in neurons. Physiol. Rev..

[131] Fliegner K.H., Ching G.Y., Liem R.K. (1990). The predicted amino acid sequence of alpha-internexin is that of a novel neuronal intermediate filament protein. EMBO J..

[132] Brownlees J., Ackerley S., Grierson A.J., Jacobsen N.J., Shea K., Anderton B.H., Leigh P.N., Shaw C.E., Miller C.C. (2002). Charcot-Marie-Tooth disease neurofilament mutations disrupt neurofilament assembly and axonal transport. Hum. Mol. Genet..

[133] Kimelberg H.K. (1981). Active accumulation and exchange transport of chloride in astroglial cells in culture. Biochim. Biophys. Acta.

[134] Kimelberg H.K., Katz D.M. (1986). Regional differences in 5-hydroxytryptamine and catecholamine uptake in primary astrocyte cultures. J. Neurochem..

[135] Kimelberg H.K., Pelton E.W.N. (1983). High-affinity uptake of [3H]norepinephrine by primary astrocyte cultures and its inhibition by tricyclic antidepressants. Neurochem. Int..

[136] Walz W., Wuttke W., Hertz L. (1984). Astrocytes in primary cultures: Membrane potential characteristics reveal exclusive potassium conductance and potassium accumulator properties. Brain Res..

[137] Syková E., Chvátal A. (1993). Extracellular ionic and volume changes: The role in glia-neuron interaction. J. Chem. Neuroanat.

[138] Parpura V., Basarsky T.A., Liu F., Jeftinija K., Jeftinija S., Haydon P.G. (1994). Glutamate-mediated astrocyte-neuron signalling. Nature.

[139] Bezzi P., Gundersen V., Galbete J.L., Seifert G., Steinhäuser C., Pilati E., Volterra A. (2004). Astrocytes contain a vesicular compartment that is competent for regulated exocytosis of glutamate. Nat. Neurosci..

[140] Zhang Q., Pangrsic T., Kreft M., Krzan M., Li N., Sul J.Y., Halassa M., Van Bockstaele E., Zorec R., Haydon P.G. (2004). Fusion-related release of glutamate from astrocytes. J. Biol. Chem..

[141] Baertschi A.J., Monnier D., Schmidt U., Levitan E.S., Fakan S., Roatti A. (2001). Acid prohormone sequence determines size, shape, and docking of secretory vesicles in atrial myocytes. Circ. Res..

[142] Krzan M., Stenovec M., Kreft M., Pangrsic T., Grilc S., Haydon P.G., Zorec R. (2003). Calcium-dependent exocytosis of atrial natriuretic peptide from astrocytes. J. Neurosci..

[143] Kreft M., Stenovec M., Rupnik M., Grilc S., Krzan M., Potokar M., Pangrsic T., Haydon P.G., Zorec R. (2004). Properties of Ca(2+)-dependent exocytosis in cultured astrocytes. Glia.

[144] Coco S., Calegari F., Pravettoni E., Pozzi D., Taverna E., Rosa P., Matteoli M., Verderio C. (2003). Storage and release of ATP from astrocytes in culture. J. Biol. Chem..

[145] Martineau M., Galli T., Baux G., Mothet J.P. (2008). Confocal imaging and tracking of the exocytotic routes for D-serine-mediated gliotransmission. Glia.

[146] Taraska J.W., Perrais D., Ohara-Imaizumi M., Nagamatsu S., Almers W. (2003). Secretory granules are recaptured largely intact after stimulated exocytosis in cultured endocrine cells. Proc. Natl. Acad. Sci. USA.

[147] Schnitzer J., Franke W.W., Schachner M. (1981). Immunocytochemical demonstration of vimentin in astrocytes and ependymal cells of developing and adult mouse nervous system. J. Cell Biol..

[148] Bignami A., Raju T., Dahl D. (1982). Localization of vimentin, the nonspecific intermediate filament protein, in embryonal glia and in early differentiating neurons. *In vivo* and *in vitro* immunofluorescence study of the rat embryo with vimentin and neurofilament antisera. Dev. Biol..

[149] Lendahl U., Zimmerman L.B., McKay R.D. (1990). CNS stem cells express a new class of intermediate filament protein. Cell.

[150] Bovolenta P., Liem R.K., Mason C.A. (1984). Development of cerebellar astroglia: Transitions in form and cytoskeletal content. Dev. Biol..

[151] Pixley S.K., de Vellis J. (1984). Transition between immature radial glia and mature astrocytes studied with a monoclonal antibody to vimentin. Brain Res..

[152] Migheli A., Pezzulo T., Attanasio A., Schiffer D. (1993). Peripherin immunoreactive structures in amyotrophic lateral sclerosis. Lab. Investig..

[153] Pappolla M.A. (1986). Lewy bodies of parkinson’s disease. Immune electron microscopic demonstration of neurofilament antigens in constituent filaments. Arch. Pathol. Lab. Med..

[154] Côté F., Collard J.F., Julien J.P. (1993). Progressive neuronopathy in transgenic mice expressing the human neurofilament heavy gene: A mouse model of amyotrophic lateral sclerosis. Cell.

[155] Lee M.K., Marszalek J.R., Cleveland D.W. (1994). A mutant neurofilament subunit causes massive, selective motor neuron death: Implications for the pathogenesis of human motor neuron disease. Neuron.

[156] Beaulieu J.M., Nguyen M.D., Julien J.P. (1999). Late onset of motor neurons in mice overexpressing wild-type peripherin. J. Cell Biol..

[157] De Vos K.J., Chapman A.L., Tennant M.E., Manser C., Tudor E.L., Lau K.F., Brownlees J., Ackerley S., Shaw P.J., McLoughlin D.M. (2007). Familial amyotrophic lateral sclerosis-linked SOD1 mutants perturb fast axonal transport to reduce axonal mitochondria content. Hum. Mol. Genet..

[158] Kieran D., Hafezparast M., Bohnert S., Dick J.R., Martin J., Schiavo G., Fisher E.M., Greensmith L. (2005). A mutation in dynein rescues axonal transport defects and extends the life span of ALS mice. J. Cell Biol..

[159] Hafezparast M., Klocke R., Ruhrberg C., Marquardt A., Ahmad-Annuar A., Bowen S., Lalli G., Witherden A.S., Hummerich H., Nicholson S. (2003). Mutations in dynein link motor neuron degeneration to defects in retrograde transport. Science.

[160] LaMonte B.H., Wallace K.E., Holloway B.A., Shelly S.S., Ascaño J., Tokito M., Van Winkle T., Howland D.S., Holzbaur E.L. (2002). Disruption of dynein/dynactin inhibits axonal transport in motor neurons causing late-onset progressive degeneration. Neuron.

[161] Corbo M., Hays A.P. (1992). Peripherin and neurofilament protein coexist in spinal spheroids of motor neuron disease. J. Neuropathol. Exp. Neurol..

[162] Wong N.K., He B.P., Strong M.J. (2000). Characterization of neuronal intermediate filament protein expression in cervical spinal motor neurons in sporadic Amyotrophic Lateral Sclerosis (ALS). J. Neuropathol. Exp. Neurol..

[163] Bergeron C., Beric-Maskarel K., Muntasser S., Weyer L., Somerville M.J., Percy M.E. (1994). Neurofilament light and polyadenylated mRNA levels are decreased in Amyotrophic Lateral Sclerosis motor neurons. J. Neuropathol. Exp. Neurol..

[164] Oguri T., Inoko A., Shima H., Izawa I., Arimura N., Yamaguchi T., Inagaki N., Kaibuchi K., Kikuchi K., Inagaki M. (2006). Vimentin-Ser82 as a memory phosphorylation site in astrocytes. Genes Cells.

[165] Laronne A., Rotkopf S., Hellman A., Gruenbaum Y., Porter A.C., Brandeis M. (2003). Synchronization of interphase events depends neither on mitosis nor on cdk1. Mol. Biol. Cell.

[166] Broers J.L., Machiels B.M., Kuijpers H.J., Smedts F., van den Kieboom R., Raymond Y., Ramaekers F.C. (1997). A- and B-type lamins are differentially expressed in normal human tissues. Histochem. Cell Biol..

[167] Gruenbaum Y., Foisner R. (2015). Lamins: Nuclear intermediate filament proteins with fundamental functions in nuclear mechanics and genome regulation. Annu. Rev. Biochem..

[168] Stuurman N., Heins S., Aebi U. (1998). Nuclear lamins: Their structure, assembly, and inteactions. J. Struct. Biol..

[169] Thompson L.J., Bollen M., Fields A.P. (1997). Identification of protein phosphatase 1 as a mitotic lamin phosphatase. J. Biol. Chem..

[170] Aubin J.E., Osborn M., Franke W.W., Weber K. (1980). Intermediate filaments of the vimentin-type and the cytokeratin-type are distributed differently during mitosis. Exp. Cell Res..

[171] Jones J.C., Goldman A.E., Yang H.Y., Goldman R.D. (1985). The organizational fate of intermediate filament networks in two epithelial cell types during mitosis. J. Cell Biol..

[172] Rosevear E.R., McReynolds M., Goldman R.D. (1990). Dynamic properties of intermediate filaments: Disassembly and reassembly during mitosis in Baby Hamster Kidney cells. Cell Motil. Cytoskelet..

[173] Berlin R.D., Oliver J.M. (1980). Surface functions during mitosis. II. Quantitation of pinocytosis and kinetic characterization of the mitotic cycle with a new fluorescence technique. J. Cell Biol..

[174] Berlin R.D., Oliver J.M., Walter R.J. (1978). Surface functions during mitosis I: Phagocytosis, pinocytosis and mobility of surface-bound con A. Cell.

[175] Tuomikoski T., Felix M.A., Dorée M., Gruenberg J. (1989). Inhibition of endocytic vesicle fusion *in vitro* by the cell-cycle control protein kinase cdc2. Nature.

[176] Boucrot E., Kirchhausen T. (2007). Endosomal recycling controls plasma membrane area during mitosis. Proc. Natl. Acad. Sci. USA.

[177] Schweitzer J.K., Burke E.E., Goodson H.V., D’Souza-Schorey C. (2005). Endocytosis resumes during late mitosis and is required for cytokinesis. J. Biol. Chem..

[178] Lanzetti L., Margaria V., Melander F., Virgili L., Lee M.H., Bartek J., Jensen S. (2007). Regulation of the Rab5 GTPase-activating protein RN-tre by the dual specificity phosphatase Cdc14A in human cells. J. Biol. Chem..

[179] Furuya T., Kim M., Lipinski M., Li J., Kim D., Lu T., Shen Y., Rameh L., Yankner B., Tsai L.H. (2010). Negative regulation of VPS34 by Cdk mediated phosphorylation. Mol. Cell.

[180] Schu P.V., Takegawa K., Fry M.J., Stack J.H., Waterfield M.D., Emr S.D. (1993). Phosphatidylinositol 3-kinase encoded by yeast VPS34 gene essential for protein sorting. Science.

[181] Simonsen A., Lippé R., Christoforidis S., Gaullier J.M., Brech A., Callaghan J., Toh B.H., Murphy C., Zerial M., Stenmark H. (1998). EEA1 links PI(3)K function to Rab5 regulation of endosome fusion. Nature.

[182] Bergeland T., Haugen L., Landsverk O.J., Stenmark H., Bakke O. (2008). Cell-cycle-dependent binding kinetics for the early endosomal tethering factor EEA1. EMBO Rep..

[183] Dyer N., Rebollo E., Dominguez P., Elkhatib N., Chavrier P., Daviet L., Gonzalez C., Gonzalez-Gaitan M. (2007). Spermatocyte cytokinesis requires rapid membrane addition mediated by ARF6 on central spindle recycling endosomes. Development.

[184] Schweitzer J.K., D’Souza-Schorey C. (2002). Localization and activation of the ARF6 GTPase during cleavage furrow ingression and cytokinesis. J. Biol. Chem..

[185] Wilson G.M., Fieling A.B., Simon G.C., Yu X., Andrews P.D., Hames R.S., Frey A.M., Peden A.A., Gould G.W., Prekeris R. (2005). The FIP3-Rab11 protein complex regulates recycling endosome targeting to the cleavage furrow during late cytokinesis. Mol. Biol. Cell.

[186] Morita E., Sandrin V., Chung H.Y., Morham S.G., Gygi S.P., Rodesch C.K., Sundquist W.I. (2007). Human ESCRT and ALIX proteins interact with proteins of the midbody and function in cytokinesis. EMBO J..

[187] Miserey-Lenkei S., Couedel-Courteille A., Del Nerv E., Bardin S., Piel M., Racine V., Sibarita J.B., Perez F., Bornens M., Goud B. (2006). A role for the Rab6a′ GTPase in the inactivation of the Mad2-spindle checkpoint. EMBO J..

[188] Kouranti I., Sachse M., Arounche N., Goud B., Echard A. (2006). Rab35 regulates an endocytic recycling pathway essential for the terminal steps of cytokinesis. Curr. Biol..

[189] Audhya A., Desai A., Oegema K. (2007). A role for Rab5 in structuring the endoplasmic reticulum. J. Cell Biol..

[190] Pellinen T., Tuomi S., Arjonen A., Wolf M., Edgren H., Meyer H., Grosse R., Kitzing T., Rantala J.K., Kallioniemi O. (2008). Integrin trafficking regulated by Rab21 is necessary for cytokinesis. Dev. Cell.

